# Stimming in a Reverie: A Case of Maladaptive Daydreaming Previously Diagnosed as Autism

**DOI:** 10.1155/crps/9700504

**Published:** 2025-03-23

**Authors:** Eli Somer, Nirit Soffer-Dudek

**Affiliations:** ^1^School of Social Work, University of Haifa, Haifa 3103301, Israel; ^2^Department of Psychology, Ben-Gurion University of the Negev, Beersheba 8443944, Israel

**Keywords:** autism spectrum disorder, case study, maladaptive daydreaming, stereotypies, stimming

## Abstract

This case study investigates the complex interplay between autism spectrum disorder (ASD) and maladaptive daydreaming (MD), focusing on the misinterpretation of stereotypical movements. The case investigates Liam, a 23-year-old male diagnosed with ASD in childhood. He sought reassessment due to suspicions that his “stimming” behaviors might be linked to MD rather than autism. We analyze Liam's freely reported experiences, self-reported scale scores, and the outcome of clinician-administered diagnostic interviews scored independently by two clinicians. Findings reveal that social communication problems were not present, negating the diagnosis of ASD, and behaviors previously attributed to ASD could be better construed as kinesthetic components of MD episodes. This case highlights the potential for misidentification of MD as ASD, mainly when stereotypical movements are present. The case study underscores the significance of awareness to MD in research and clinical settings. It also illuminates the critical importance of differential diagnosis in cases of ASD, as similar behavioral manifestations may stem from distinct underlying conditions. This study contributes to the emerging body of literature on the relationship between ASD and MD and calls for increased awareness among clinicians about the potential overlap in symptoms between these conditions. We discuss future research directions and implications for clinical practice.

## 1. Introduction

This case study examines Liam, a 23-year-old man diagnosed with autism spectrum disorder (ASD) during his childhood. He sought reassessment, suspecting that his previously identified autism-related stereotypical movements (stimming) might be a kinesthetic component of maladaptive daydreaming (MD). This case demonstrates the importance of differential diagnosis in ASD, as similar behaviors may stem from distinct conditions. Accurate diagnosis can lead to more appropriate treatments, potentially improving well-being and functioning.

MD, initially also known as “compulsive fantasy”, is a proposed psychological disorder characterized by excessively engaging in vivid, narrative, and intensely emotional fantasy activities to the extent that it causes clinically significant distress and/or functional impairment [1]. This condition is marked by compulsive daydreaming frequently triggered by repetitive movements or music, leading to substantial disruption in daily life [2]. Research indicates that MD is not merely a common experience but a distinct clinical condition, with a point-prevalence of 2.5% in the general population, similar to other internalizing psychiatric syndromes [3]. While MD is not yet recognized as a formal disorder, it is associated with heightened levels of ADHD, anxiety disorders, depressive disorders, and OCD spectrum disorders [4], as well as a heightened risk of suicidality [5].

Recent research has highlighted the complex relationship between ASD and MD, suggesting that these conditions may co-occur more frequently than previously thought. MD and motor stereotypy in ASD share significant overlaps, characterized by repetitive movements and immersive mental imagery [6] Clinical evidence shows that both MD and motor stereotypy in ASD involve repetitive kinesthetic activity that is often triggered by strong emotions. Individuals with these conditions report entering trance-like states during their episodes. Studies have found that a significant proportion of adults with ASD report experiences consistent with MD, challenging earlier assumptions about limited imaginative capacities in individuals with ASD. In fact, in an ASD sample, 42% reported elevated MD scores [6]. As West et al. [7] have noted, the intersection of ASD and MD presents a unique clinical picture. Individuals experiencing both conditions may face elevated levels of loneliness and emotion regulation challenges compared to those with either condition alone. Furthermore, the repetitive behaviors associated with ASD may serve to deepen absorption in daydreaming for those who also experience MD. While MD is not currently considered a clinical feature of ASD, there is evidence of high rates of MD symptoms in ASD populations and vice versa [7].

In a paper on stereotypical movement disorder (SMD), Freeman et al. [8] argued that differentiation of SMD from tics and ASD is crucial to avoid misdiagnosis and unnecessary treatment. They reported that children (but not their parents) liked their stereotypies, which were usually associated with excitement or imaginative play. Recent evidence demonstrating the connection between childhood motor stereotypy and daydreaming was also provided by Hedderly et al. [9], who showed an association between childhood complex motor stereotypies and adult-reported MD.

Although at times they may seem similar to an outside observer, MD and ASD essentially differ. The main symptom in MD, namely, excessive, addictive fantasy, is not necessarily present in ASD. ASD is defined first and foremost by the critical element of impairments in social communication or social understanding, which is not a part of MD criteria and does not seem to characterize most individuals with MD. Typically, when individuals with MD face social difficulties, they stem more from a preference for daydreaming over socializing, rather than from an inherent social skill deficit condition like ASD. The second defining feature of ASD, according to the DSM-5-TR [10], is restricted repetitive patterns of behavior, activities, or interests, which can include, among other things, a remarkable interest in one's inner world, which is central to MD, and unusual kinesthetic properties, which often characterize MD as well. However, the nature of repetitive movements also differs between MD and ASD. Individuals with MD consciously perform movements predominantly not only to induce or maintain the daydreaming state but also to embody their fantasized actions [11]. They may describe the movements and daydreaming as an itch that needs to be scratched. For example, one person with MD shared that: “I have to pace when I'm dreaming even if my feet ached or if it is two in the morning and I'm already in bed. I toss something in my hand and flap my hands” [1]. People with MD often report that they avoid publicly performing the movements so they can daydream the most intensely only when alone.

Conversely, those with ASD are said to report unconscious movements as outputs of internal processes for which they have limited awareness and control [7]. For example, a person with Autism shared that stereotyped movements are not willed for any specific reason but are just “things that happen by themselves when I'm not paying attention to my body” [12]. He found it hard to describe what it feels like to engage in the movements because, he explained, if he pays attention to them, they cease. Like people with MD, the patient described learning to monitor and control these movements in public. However, if he is “…worn out, distracted, overwhelmed, intensely focused on something else, or just relaxed and off-guard”, the monitoring would fail, and the movements would occur [12]). This observational overlap raises essential questions about diagnosis, intervention strategies, and the broader understanding of repetitive behaviors in neurodevelopmental disorders, highlighting the need for further research to distinguish between MD and motor stereotypy in ASD and develop appropriate clinical approaches [13].

This case provides an opportunity to explore the nuanced presentation of ASD and MD symptoms, highlighting the importance of careful assessment and the need to consider the possibility of rich imaginative experiences in individuals with ASD or ASD-like symptoms. This highlights the challenge of distinguishing ASD behaviors from those potentially related to MD, especially when social communication difficulties are less apparent.

## 2. Method

### 2.1. Assessment Tools

#### 2.1.1. Documents Supporting the Childhood Diagnosis of ASD

To verify Liam's earlier ASD diagnosis, we reviewed the following supporting documents: Reports by his kindergarten teacher and an occupational therapist outlining concerns about the child's extensive stereotypical movements, tendency to retreat into daydreaming, and social withdrawal; A National Insurance Institute diagnostic report confirming a diagnosis of Asperger Syndrome; A National Insurance Institute approval letter for a 100% Child Disability Allowance due to Autism; A document of discharge from Israel's mandatory military service on the grounds of “medical incompatibility.”

#### 2.1.2. Self-Report Scales

##### 2.1.2.1. The 16-item Maladaptive Daydreaming Scale

The 16-item Maladaptive Daydreaming Scale (MDS-16; [4] is an expanded version of the original 14-item Maladaptive Daydreaming Scale [14], which measures characteristics of MD across four components with high internal consistency: Yearning (e.g., “Some people feel a need to continue a daydream that was interrupted by a real-world event at a later point. When a real-world event has interrupted one of your daydreams, how strong was your need or urge to return to that daydream as soon as possible?”), Kinesthesia (e.g., “How often do your current daydreams accompany exercise like walking, swinging, or shaking hands?”), Impairment (e.g., “Some people have the experience of their daydreaming interfering with their daily chores or tasks. How much does your daydreaming interfere with your ability to get basic chores accomplished?”), and relation to music (e.g., “Some people find it hard to maintain their daydreaming when they are not listening to music. To what extent is your daydreaming dependent on continued listening to music?”). Participants are asked to respond to each item using a scale ranging from 0% (never) to 100% (extremely frequent), with 10% increments. We used a cutoff score of 40 to differentiate maladaptive from non-MD, as recommended in previous research [15]. This study used the Hebrew version of the MDS-16 [16].

##### 2.1.2.2. The Autism Spectrum Quotient

The Autism Spectrum Quotient (AQ, [17]) is a widely used self-report questionnaire to assess autistic traits in adults. The AQ consists of 50 items that gauge the degree to which individuals exhibit characteristics associated with the autism spectrum. It is designed for adults aged 16 and older who possess normal intelligence. The AQ is structured around five key domains, each containing 10 items: Social Skills, Attention Switching, Attention to Detail, Communication, and Imagination. Respondents rate each item on a 4-point scale, from “definitely agree” to “definitely disagree.” Notably, some items are reverse scored, meaning a higher score reflects lower autistic traits. The total AQ score can range from 0 (indicating no autistic traits) to 50 (indicating maximum ASD traits) [17]. The AQ has undergone extensive validation to assess its psychometric properties.

Research suggests that the AQ is effective in distinguishing between individuals with ASD and those without it, demonstrating adequate sensitivity and specificity. The original recommended cutoff was 32 [17], while later research identified a lower cutoff of 26 [18]. The AQ has shown good internal consistency, with Cronbach's alpha values typically exceeding the acceptable threshold of 0.70. The AQ has demonstrated usefulness in non-English speaking samples (e.g., [19]). The first author and his team translated AQ into Hebrew using a collaborative and iterative method [20]. This approach emphasizes collaboration among translators and researchers. It involves multiple iterations of translation and feedback, allowing for adjustments based on cultural context and respondent understanding. This method ensures that the translated instrument accurately reflects the intended meaning rather than just a literal translation.

#### 2.1.3. Clinical Interviews

##### 2.1.3.1. The Structured Clinical Interview for Maladaptive Daydreaming

The Structured Clinical Interview for Maladaptive Daydreaming (SCIMD) was developed based on the proposed diagnostic criteria for MD [21]. It is administered by a clinician or trained mental health professional familiar with MD diagnostic criteria. The SCIMD consists of a 10-question probe (and subsequent additional follow-up questions) for inclusion criteria and one probe for an exclusion criterion (and its follow-up questions). A diagnosis of MD is made if participants respond affirmatively to questions about two or more of the inclusion criteria and the differential diagnosis exclusion criterion (“not due to the direct physiological effects of a substance or a general medical condition”). The SCIMD demonstrated good inter-rater reliability and excellent agreement with a self-report measure for the disorder [21]. The first author and his team translated SCIMD into Hebrew using a collaborative and iterative method [20]. The Hebrew version has been used in previous studies (e.g., [3]).

##### 2.1.3.2. DSM-5 Interview for ASD

We administered a semistructured diagnostic ASD interview (DSM-5 ASD interview, [22] to evaluate former and current traits and features of Autism. This semistructured interview is based on the criteria of the DSM-5 for ASD and has previously been used in other studies (e.g., [23, 24]). The authors collaboratively translated the DSM-5 ASD interview into Hebrew.

##### 2.1.3.3. Anamnestic Interview

We also conducted an in-depth anamnestic interview to gather comprehensive background information on Liam's life and the history of the presenting problem. This interview was foundational in understanding the patient's personal, familial, and psychosocial context and the symptoms' onset, development, and evolution. The semistructured interview allowed for open-ended exploration and focused inquiry into specific areas of interest. The interview began with questions about his early life, including childhood experiences, family dynamics, and significant relationships. It included probing into any history of trauma, abuse, or neglect. We asked Liam about his educational journey, professional achievements, and challenges he faced in these areas. We focused on academic or professional difficulty periods that might correlate with the symptoms. We also explored his social network, including friendships, romantic relationships, and community involvement. Liam provided details of any past medical conditions and history of substance use, including alcohol, tobacco, or any other drugs. A thorough exploration of any previous psychiatric diagnoses, treatments, and hospitalizations was conducted. We also asked the interviewee about any history of psychotropic medication use, including current prescriptions and adherence.

Liam was encouraged to describe the symptoms in detail, including their onset, duration, and frequency. Questions were asked to understand any potential triggers or exacerbating factors. The interview sought to assess the impact of the symptoms on various domains of the patient's life, including work, relationships, and daily activities. The interviewee's coping strategies and any attempts at self-management were also discussed. Information was also gathered regarding prior attempts to address the presenting problem, including previous therapeutic interventions.

We also inquired about any current financial, occupational, or interpersonal stressors. The interview explored how these stressors may be contributing to the presenting problem. Other interview questions focused on the availability and quality of Liam's support systems, including family, friends, and community resources.

We documented the respondent's responses verbatim where relevant, ensuring an accurate and detailed account of their history and presenting problem.

#### 2.1.4. Observational Data

Liam directed us to a YouTube video where he demonstrates and explains his self-stimulatory behavior-induced daydreaming, which he termed “stimming” [25]. This visual information bolstered the behavioral data we obtained while observing Liam's verbal and interpersonal conduct during our interviews.

### 2.2. Procedure

Given the considerable geographical distances between the interviewee's location and the interviewers, we conducted the structured clinical interviews via video conference. The first author (ES) conducted the DSM-5 ASD interview directly with the participant, while the second author (NSD) observed and took detailed notes. For the SCIMD, the authors switched roles. To ensure the reliability of the analysis, both authors independently reviewed, analyzed, and scored the data from both interviews. After completing our analyses, we compared our findings to evaluate the degree of agreement. The results were unanimous, demonstrating high consistency in our interpretations.

#### 2.2.1. Ethical Considerations

In conducting this case study, we maintained rigorous ethical standards throughout the research process. The study received prior approval from the Faculty of Social Welfare and Health Sciences Research Ethics Committee at the University of Haifa (Certificate No. 239/24), ensuring adherence to institutional guidelines for research involving human participants.

The interviewee provided informed consent for both his assessment and his publication, fully understanding the purpose of the study and the nature of his involvement. The final report anonymized or altered all identifying information to protect the participant's privacy. Additionally, we provided the interviewee with the final manuscript for review and confirmed that the data accurately reflected his experiences. We conducted a debriefing interview postdata collection to assess and ensure the participant's emotional well-being, reflecting our commitment to minimizing potential psychological harm. Additionally, the participant was given our contact information and encouraged to reach out with any questions or concerns that might arise, further emphasizing our dedication to ongoing ethical responsibility.

## 3. Findings

### 3.1. Structured Clinical Interviews

Table 1contains scoring for both structured interviews (ASD and MD) by both clinician-researchers. As can be seen in the table, scoring was nearly identical, with the same bottom line: Both of us found Liam to be positive for MD and negative for ASD because there was no evidence for social communication problems. We based the latter finding on the content of Liam's reports during the interview, informing us that he had no specific problems in making or maintaining social connections or in understanding sarcasm or undertones, no tendency to offend people accidentally, that he never had any particular issues with eye contact or facial expressions and does not avoid social assessments, etc. We corroborated Liam's self-reports with our clinical observations during the assessment interviews. The interviewee was very much attuned to our questions, understood their nuances, used appropriate body language and nonverbal signals, and answered in a relevant fashion. In short, he was able to communicate with us very well. The results of the structured clinical interviews are presented in Table 1.

### 3.2. Self-Report Scales

On the MDS-16, Liam received a mean score of 78.13, well above the clinical cutoff of 40. On individual items, with the scale ranging from 0 to 100, all his answers ranged between 50 and 100, except for item 16, which he answered 0, indicating that his daydreaming is not necessarily contingent upon listening to music.

On the AQ, Liam received a total score of 14, well under the clinical cutoff of 26 [18]. In the original validating study on the AQ, the Asperger's/high functioning Autism group had an average of 35.8, whereas the random control group had an average of 16.4 [17]. In that study, the suggested clinical cutoff was even higher, namely, 32.

Thus, the self-report scales coincide with the results of the clinical interviews, again suggesting that Liam suffers from MD but not ASD.

### 3.3. Reported History, Symptoms, and Motives for Participation in the Study

Besides his formal diagnosis, Liam had recently believed he was autistic based on observed similarities between his behaviors and those of people with autism. After Googling his symptoms, 23-year-old Liam approached the researchers known for their work on MD. He had been experiencing lifelong, extensive, vivid daydreaming accompanied by rhythmic, repetitive movements, and sounds resulting from mumbling the contents of his fantasies. The movements included fast pacing, slight jumping, and shaking his hands or handheld objects, usually of an elastic nature, such as a shoelace or a leaf. Liam describes the movements as a byproduct of the imaginative activity, enabling the state of total immersion and detachment from the physical realm. Although he may also feel an urge to shake the object when not daydreaming (mainly when concentrating on something interesting in a socially safe environment), the activity is most pronounced during daydreaming. Such concentration with object-shaking often brings about a full-blown daydreaming episode. Only after reading about MD did Liam realize that purposeful stereotypical movements are not exclusive to people with ASD. He began to question his previous diagnosis of ASD and sought more information. Liam reported tracing his movement and daydreaming behaviors to age 5 when his idiosyncratic behavior alarmed his kindergarten teacher, prompting the first neurodevelopmental assessment. At age 6, Liam learned to hide his “stimming” to avoid social scrutiny from adults and peers. Since then, he has felt “different,” recognizing that others did not share his favorite pastime. Documents Liam shared with us revealed that despite some clinical doubts, he was diagnosed with Asperger Syndrome, later reclassified as ASD. Liam's developmental assessment revealed sensory processing, fine motor skills, and organization issues, leading to an occupational therapy referral. While the complete assessment data were no longer available, we learned from Liam that these were mild difficulties that improved with intervention, suggesting these challenges may have been related to developmental delays rather than core features of ASD. The diagnostician observing Liam in kindergarten noted his ability to connect with peers and his tendency to retreat to quieter spaces for repetitive motions and self-absorption. During his time in kindergarten, Liam did respond positively to his peers' attempts to interact with him and would occasionally initiate age-appropriate imaginative play with other children. In retrospect, Liam reported that at this early age, he did not experience the self-consciousness about his daydreaming that typically characterizes MD in older individuals. His kindergarten peers appeared to accept his periodic withdrawal and stimming behaviors without comment or apparent judgment. This lack of self-consciousness about his behaviors stands in contrast to his later experiences, when awareness of social stigma led him to carefully conceal his daydreaming activities

Liam's diagnosis led to 100% disability status and financial compensation for Autism from the National Insurance Institute from early childhood up to the age of 18. It also exempted him from compulsory military service at age 18. Liam never received psychotherapy or any psychiatric medication and never used any recreational or illicit drugs.

Liam estimated that he currently engages in stimming-generated daydreaming about 10 times a day, with 20-minute episodes accumulating to more than three daily hours dedicated to daydreaming. Liam reports experiencing distress and restlessness when unable to daydream. He acknowledges that many lifestyle decisions were influenced by the desired freedom to daydream unhindered. For example, he was pleased with his discharge from mandatory service in the Israel Defense Forces and chose not to volunteer because he would not have the privacy to daydream if he served. His daydreaming episodes may be triggered by various stimuli such as reading a book or reading something online, watching a particular TV show, or exciting video clip on social media, random thoughts Liam may have during the day or at bedtime, or an interesting idea arising from a conversation with someone (in which case he will try to postpone or avoid succumbing to the urge to fantasize). Notably, Liam never inserts himself into these storylines. Instead, he draws inspiration from prominent figures such as famous politicians, YouTube influencers, musicians, or singers. After studying their biographies, he elaborates on them and diverges from these initial real-life figures to create his fantasized protagonists. When creating new fantasy characters, Liam often combines a real person's appearance with a fantasized personality. He then enjoys imagining these characters' speeches, career decisions, stage performances, and life events. He also internally “watches” videos created by these imagined YouTube influencers and envisions concerts and original music performed by imagined artists, even though he is not a musician.

### 3.4. Participant Background and Functioning Level

Despite this potentially distracting condition, Liam completed high school and passed his matriculation exams. He described himself as a friendly boy who enjoyed his friends' company and still does today—although he also enjoys spending time alone. When asked about long-lasting friendships, Liam described maintaining relationships from middle school, spanning 8–9 years. He reported an ability to have meaningful, intimate conversations with close friends. Liam mentioned that in some new social situations, he may feel uncomfortable and become shy or silent, but in others, he can feel comfortable and act naturally. Liam's context-dependent social comfort and his ability to interact naturally in familiar settings suggests that his social hesitancy stems from limited social exposure due to MD-related isolation, rather than the pervasive social communication difficulties typical of ASD.

Although he made several courting attempts, Liam never had a girlfriend. Liam spent a year living with roommates while studying for a 2-year college degree. Still, in the second year, he moved to his own rented apartment because the lack of privacy made it difficult for him to daydream. He graduated despite having trouble studying due to his daydreaming. He reports still experiencing such difficulties when he attempts to learn and develop further in his field. He lives in his apartment in a small village where his parents and grandmother reside. Liam receives some financial support from his parents, which is common for his age in Israel. Otherwise, he is fully functional, working as a freelancer in a creative field and managing his household independently (e.g., cooking, doing his laundry, etc.).

### 3.5. Participant's Perspective on his Symptoms

Liam describes this activity as a liberating comfort zone that is never boring. However, he feels distressed by the thought of wasting time, not necessarily because of the amount of time dedicated to the activity, but more so because of its quality, namely, his tendency to repeat fantasies or focus on topics he considers unimportant (Liam compares this to watching “trashy” shows on TV instead of an educational documentary). He wishes to navigate his daydreaming to more creative avenues. Besides this content-related problem, he believes daydreaming impairs his academic performance, as he is drawn to it instead of studying efficiently. While he wants more control over this activity, he does not want to eliminate it. Aligning with neurodiversity advocates who oppose medical efforts to eradicate Autism [26], Liam believes stimming should not be viewed as a physical illness to be cured. His YouTube video and participation in this study are motivated by his goal to promote acceptance, accommodation, and support for “neuro-minorities” in society. Like proponents of neurodiversity, Liam considers it harmful to view stimming solely as a problem to be fixed.

## 4. Discussion

The case of Liam, a young man initially diagnosed with ASD but later identified as experiencing MD, offers a compelling lens through which to examine the complexities of differential diagnosis in neurodevelopmental and psychological disorders. This case study unveils several intriguing theoretical concepts that contribute to our current understanding of the interrelationship between these conditions.

At the heart of this case lies the notion of a spectrum of immersive inner experiences that may span across different diagnostic categories. Liam's experiences suggest that the boundaries between typical daydreaming, MD, and some ASD-related experiences might be more fluid than previously thought. The overlap in behavioral manifestations, particularly stereotypical movements, hints at shared neuropsychological mechanisms underlying these conditions. This observation challenges the prevailing categorical approach to diagnosis and points towards a more dimensional understanding of these experiences. Such a perspective could help shape how we conceptualize and diagnose conditions characterized by rich inner lives and atypical behaviors.

The role of kinesthetic components in fantasy engagement emerges as another crucial insight from Liam's case. The repetitive movements, traditionally associated with ASD “stimming,” were revealed to be instrumental in inducing and sustaining Liam's daydreaming state. This finding suggests that motor activity might play a more significant role in fantasy and imagination than previously recognized [11], not just in MD but potentially in other conditions characterized by vivid inner experiences. This connection between physical movement and mental imagery opens new avenues for understanding the embodied nature of cognition and imagination.

From childhood to young adulthood, Liam's developmental trajectory provides valuable insights into the life course of individuals with pronounced fantasy tendencies. His story suggests that these propensities may manifest early in life and persist, evolving in complexity and impact over time. This developmental perspective is crucial for understanding how conditions like MD might be misdiagnosed or overlooked in childhood and how they might interact with social and educational experiences throughout development. Research on adults reveals that MD can be improved through targeted interventions [27]. Although research on treating children with MD is lacking, it is reasonable to assume that correctly identifying the child's symptoms and intervening accordingly is crucial for improving the child's well-being. Liam could have benefitted from earlier psychoeducation as well as psychotherapeutic interventions, such as helping him monitor and control his symptoms while still being able to enjoy them. Liam's case underscores the importance of early identification and support of children with rich, addictive fantasy lives and the understanding of how these experiences may shape their social development.

The misdiagnosis of ASD in Liam's case highlights the profound impact that diagnostic labels can have on an individual's self-concept, life choices, and treatment trajectory. This misdiagnosis shaped Liam's educational experiences, social interactions, and even his exemption from a compulsory military service. Specifically, Liam's early diagnosis of ASD led his family and teachers to overly focus on his unusual behaviors and social deficits, rather than acknowledging and supporting his vivid inner imaginative world. Occasionally, his peculiar demeanor and disengagement frustrated family and classmates, resulting in negative feedback that marginalized him. Liam's family and peers struggled to comprehend his intense daydreaming tendencies and apparent lack of interest in others, often labeling him as “odd,” which hindered any efforts to develop meaningful relationships. In addition to the negative effects of the inaccurate ASD diagnosis, Liam's undiagnosed and untreated MD also impaired his attentional resources, making it difficult for him to concentrate on his academic pursuits. This attention deficit, compounded by the allure of his gratifying fantasy life, ultimately led to his decision to drop out of college and secure only modest employment.

Liam's academic, social, and occupational impairments highlight the potential long-term consequences of misdiagnosis, primarily when it occurs in early childhood. This aspect of the case calls for a more nuanced approach to the diagnosis of ASD, particularly in individuals who exhibit stimming behaviors and a rich fantasy life. This case has implications beyond the interviewee's perspective, extending to a broader social level. ASD is a highly debilitating condition that often requires expensive support, such as long-term psychotherapy and the employment of a teacher's aide [28]. Parents of children with ASD may need to reduce their work hours, which is why, in Israel, the diagnosis entitles families to substantial disability payments from the state. In contrast, in our clinical experience, we found that MD generally impairs functioning to a lesser extent, similar to common conditions like anxiety or depression. Accurately differentiating between ASD and MD could help direct the taxpayer funds to where they are most needed. Further research is needed to examine the scale of this problem.

While this case study provides rich insights, it is essential to acknowledge its limitations. As a single case study, the generalizability of findings is limited. Liam's experiences may not be representative of all individuals with MD or those misdiagnosed with ASD. The retrospective nature of some of the data, relying on Liam's recollections of childhood experiences, introduces the possibility of recall bias. Furthermore, the lack of blinding in the study may have introduced bias among the researcher-interviewers. Finally, the study's situatedness within a particular cultural context may limit its applicability to other cultural settings where ASD and MD may be conceptualized differently.

Nevertheless, this case study opens up several exciting avenues for future research. There is a clear need for large-scale comparative studies to help delineate the boundaries and overlaps between MD, ASD, and typical development. Such studies could focus on measures of fantasy proneness, social cognition, and stereotypies. Neuroimaging research comparing brain activity patterns during daydreaming episodes in individuals with MD, ASD, and controls could provide crucial insights into the neural mechanisms underlying these experiences.

Longitudinal studies following children identified as having rich fantasy lives or excessive daydreaming tendencies could help elucidate the developmental trajectories of MD and its potential relationship to ASD. Such studies could inform early intervention strategies and support for these individuals. Additionally, developing and testing interventions designed explicitly for MD and comparing their efficacy to ASD-focused interventions could significantly enhance our therapeutic approaches for individuals who exhibit overlapping symptoms. In-depth phenomenological studies exploring the lived experiences of individuals with MD, particularly those who have been misdiagnosed with other conditions, could enhance our understanding of the subjective nature of this condition. These studies could provide rich, qualitative data to complement quantitative research efforts.

Finally, the present study provides support for the recognition of MD as a disorder in psychiatric manuals. Such recognition would increase awareness in research and clinical settings, enabling accurate differential diagnoses for children and adults. Many adults with MD have reported that current diagnostic labels inadequately capture their symptoms, leading to misunderstanding and ineffective treatment from therapists [1].

Some researchers suggest categorizing MD as a dissociative disorder due to its characteristic detachment from reality in favor of an internal world [29]. However, the present case also hints at MD's potential relevance to a motor stereotypy continuum within the context of neurodevelopmental disorders. Future studies should further explore the appropriate placement of MD within psychiatric nosography.

In conclusion, Liam's case illuminates the complex interplay between MD and ASD, highlighting the need for more nuanced approaches to understanding and treating individuals with rich inner lives and atypical behaviors. As research in this area progresses, it is crucial to balance recognizing the potential impairments associated with excessive daydreaming and respecting the value that some individuals place on these experiences as part of their identity and cognitive style. This case study catalyzes rethinking our approaches to diagnosis and support for individuals who fall into these complex and often overlapping categories of neurodevelopmental and psychological experiences.

## Figures and Tables

**Table 1:** Independent scoring by both clinicians on the two structured clinical interviews for (1) autism spectrum disorder (ASD) and (2) maladaptive daydreaming (MD).

Diagnostic criterion			Interviewer 1 (ES)	Interviewer 2 (NSD)
ASD	(A) Social communication	A1	No	No
		A2	No	No
		A3	No	No
	(B) Restrictive behaviors	B1	Yes (Stereotyped, repetitive movements, use of objects)	Yes (“Stimming,” and perhaps ordering toys in childhood)
		B2	No	No
		B3	Yes (fixation on rappers, specific musicians, specific TV series, YouTube/TikTok clips)	Yes (daydreaming with movements can be considered a fixed, unusual interest/preoccupation)
		B4	Yes (hyporeactivity to hunger signals)^a^	No
	(C) Symptoms present in early development	C	Yes	Yes
	(D) Impairment	D	Yes	Yes
	(E) No intellectual disability	E	Yes	Yes
	ASD diagnosis and severity rating		Negative for A, Positive for B. Negative for ASD. Severity: No level is appropriate. Does not need support at all for either A or B. Is independent and functional	Negative for A, Positive for B. Negative for ASD. Severity: No level is appropriate. Does not need support at all for either A or B. Is independent and functional

MD	(A) Persistent, vivid fantasy activity over the past 6 months	A1	Yes	Yes
		A2	Yes	Yes
	(B) Characteristics of daydreaming	B1	Yes	Yes
		B2	No	No
		B3	Yes	Yes
		B4	Yes	Yes
		B5	Yes	Yes
		B6	Yes	Yes
		B7	Yes	Yes
		B8	Yes	Yes
	(C) Impairment	C	Yes	Yes
	(D) Not due to substance	D	Yes (not explained by substance)	Yes (not explained by substance)
	MD diagnosis and severity rating		Meets criteria for MD. Severity: Moderate (one field of functioning negatively affected, namely, academic studies)	Meets criteria for MD. Severity: Moderate (one field of functioning negatively affected, namely, academic studies)

*Note*: To be positive for ASD, criteria A through E should be met. All three subcriteria (A1, A2, A3) must be positive to meet criterion A. To meet criterion B, at least two out of four are needed. To be positive for MD, criteria A through D should be met. To meet criterion A, both A1 and A2 subcriteria must be positive. To meet criterion B, at least one out of B1–B8 is needed.

^a^This potential symptom was also documented by the other reviewer (NSD) during the interview, but after consulting the DSM's exact wording, she concluded that it does not meet the criterion.

## Data Availability

The data that support the findings of this study are available on request from the corresponding author. The data are not publicly available due to privacy or ethical restrictions.
